# Strategies to Prevent Biofilm Infections on Biomaterials: Effect of Novel Naturally-Derived Biofilm Inhibitors on a Competitive Colonization Model of Titanium by *Staphylococcus aureus* and SaOS-2 Cells

**DOI:** 10.3390/microorganisms8030345

**Published:** 2020-02-29

**Authors:** Inés Reigada, Ramón Pérez-Tanoira, Jayendra Z. Patel, Kirsi Savijoki, Jari Yli-Kauhaluoma, Teemu J. Kinnari, Adyary Fallarero

**Affiliations:** 1 Drug Research Program, Division of Pharmaceutical Biosciences, Faculty of Pharmacy, University of Helsinki, 00790 Helsinki, Finland; ines.reigada@helsinki.fi (I.R.); kirsi.savijoki@helsinki.fi (K.S.); 2 Department of Otorhinolaryngology-Head and Neck Surgery, Helsinki University Hospital, University of Helsinki, Kasarmikatu 11-13, 00029 HUS, 00130 Helsinki, Finland; ramontanoira@hotmail.com (R.P.-T.); teemu.j.kinnari@helsinki.fi (T.J.K.); 3 Department of Clinical Microbiology, Hospital Universitario Príncipe de Asturias, 28805 Alcalá de Henares, Madrid, Spain; 4 Drug Research Program, Division of Pharmaceutical Chemistry and Technology, Faculty of Pharmacy, University of Helsinki, 00790 Helsinki, Finland; jayendra.patel@helsinki.fi (J.Z.P.);

**Keywords:** biofilm, co-culture, *Staphylococcus aureus*, SaOS-2, biomaterials, implanted devices

## Abstract

Biofilm-mediated infection is a major cause of bone prosthesis failure. The lack of molecules able to act in biofilms has driven research aimed at identifying new anti-biofilm agents via chemical screens. However, to be able to accommodate a large number of compounds, the testing conditions of these screenings end up being typically far from the clinical scenario. In this study, we assess the potential applicability of three previously discovered anti-biofilm compounds to be part of implanted medical devices by testing them on *in vitro* systems that more closely resemble the clinical scenario. To that end, we used a competition model based on the co-culture of SaOS-2 mammalian cells and *Staphylococcus aureus* (collection and clinical strains) on a titanium surface, as well as titanium pre-conditioned with high serum protein concentration. Additionally, we studied whether these compounds enhance the previously proven protective effect of pre-incubating titanium with SaOS-2 cells. Out of the three, DHA1 was the one with the highest potential, showing a preventive effect on bacterial adherence in all tested conditions, making it the most promising agent for incorporation into bone implants. This study emphasizes and demonstrates the importance of using meaningful experimental models, where potential antimicrobials ought to be tested for the protection of biomaterials in translational applications.

## 1. Introduction

Antimicrobial resistance is one of the major healthcare challenges that is currently faced by humanity. By switching into the biofilm state, bacteria can withstand antibiotic chemotherapy, and this is increasingly regarded as the most important nonspecific mechanism of antimicrobial resistance [1,2]. Biofilms are defined as a community of cells encased within a self-produced matrix that adheres to biological or non-biological surfaces [3,4]. Because implanted medical devices can be ideal substrates for bacteria to attach, biofilm-mediated infections are one of the leading causes of prosthesis implantation failures. Biomaterial-associated infections (BAIs) represent a great clinical concern because they cause increased morbidity and distress in patients, along with high economic costs due to increased hospitalizations [5]. 

When implanting a biomaterial, the desired outcome is the correct integration of such material with the host tissue. However, this ideal outcome is often impacted by the presence of bacterial cells at the moment of implantation. According to the concept of “race for the surface” [6], if host cells are able to colonize the surface of the device first, the chances of bacterial cells to adhere to such surface are lower, therefore lowering the risk of implant infection [7]. A frequent route of infection for implants occurs during surgery [8], as microorganisms can be introduced on the implant surface, providing them with an advantage to colonize the unprotected surface and create a biofilm [9]. Taking this into consideration, a reasonable approach would be to design an antimicrobial material or coating that promotes tissue integration. In that direction, based on the earlier results by Perez-Tanoira et al. [10], it seems that the pre-conditioning of the material with host cells is advantageous to protect against bacterial colonization. *Staphylococcus aureus* is found asymptomatically on the skin [11] and its presence there enhances the risk of infection in the surgical site, which is why it is regarded as a frequent causative agent of implant-related infections, especially in orthopedics [12].

**Figure 1:**
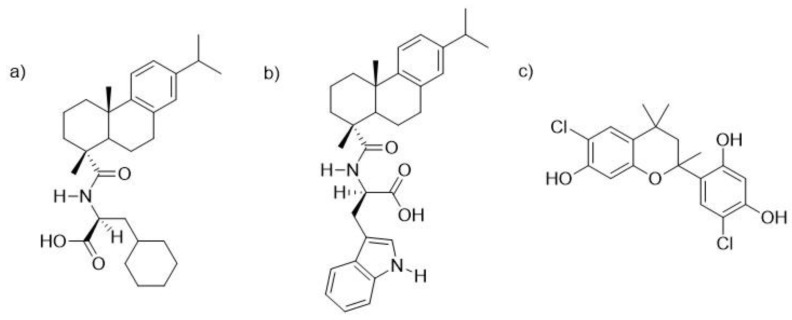
Chemical structures of the two dehydroabietic acid (DHA) derivatives, (**a**) *N*-(abiet-8,11,13-trien-18-oyl)cyclohexyl-L-alanine and (**b**) *N*-(abiet-8,11,13-trien-18-oyl)-D-tryptophan, coded **DHA1** and **DHA2**, as well as the flavan derivative (**c**), 6-chloro-4-(6-chloro-7-hydroxy-2,4,4-trimethylchroman-2-yl)benzene-1,3-diol, coded **FLA1**.

Thus far, there is a limited repertoire of compounds that are able to act on biofilms at sufficiently low concentrations, especially in the case of *S. aureus* [13]. In order to tackle this problem, several strategies have been proposed, which include, among others, (i) the screening of compounds libraries to identify new small molecules able to inhibit or disassemble biofilms [14], (ii) medicinal chemistry-driven approaches directed towards synthetic modifications of known antibiotics to increase their effectivity on biofilms [15], and (iii) development of novel nano-formulations to improve antibiotic penetration on biofilms [16]. Our laboratory has embraced the exploration of natural compound sources and those studies have resulted in the identification of three promising anti-biofilm leads [17,18,19], which are shown in Figure 1. 

The first two compounds are dehydroabietic acid (**DHA**) derivatives, *N*-(abiet-8,11,13-trien-18-oyl) cyclohexyl-L-alanine and *N*-(abiet-8,11,13-trien-18-oyl)-D-tryptophan, coded **DHA1** (shown in Figure 1a) and **DHA2** (shown in Figure 1b), respectively. These two compounds were synthetically developed in [20] (coded 11 and 9b, respectively, in [20]), by combining the abietane moiety with amino acids, which had separately been shown to display anti-biofilm properties [17,21,22,23]. Compounds **DHA1** and **DHA2** were both demonstrated to prevent biofilm formation as well as to effectively disassemble pre-formed *S. aureus* biofilms [20], and they represent the most potent abietane-type anti-biofilm agents that have been reported thus far. The third antimicrobial candidate (show in Figure 1c) is a flavan derivative: 6-chloro-4-(6-chloro-7-hydroxy-2,4,4-trimethylchroman-2-yl)benzene-1,3-diol, coded **FLA1**, that has also been earlier shown to prevent bacterial colonization and decrease the viability of existing *S. aureus* biofilms [18] (coded 291 in [18]). 

These three lead compounds are good candidates to protect medical devices from biofilm infections. Among the strategies used to protect implants are the modification of the material surfaces to inhibit bacterial adhesion [24,25] and the application of passive coatings to enhance tissue integration or compatibility. Altogether, these strategies aim at diminishing the rate of implant infection [26], which is essential not only to prevent biofilm formation per se but also to avoid aseptic loosening, which is also an important cause of prosthesis failure [27]. It is conceivable that these three lead compounds could be used to develop new materials loaded with them, or that they could be incorporated into antimicrobial coating solutions.

However, moving from *in vitro* to translational studies can be highly challenging. Often, antimicrobial screening is carried out on materials like polystyrene, which might provide dramatically different results from those obtained in more clinically-relevant surfaces, such as titanium. Similarly, in antibacterial screens, collection strains are often used, whose virulence and adherence capability might differ from those found in wild-type strains isolated from implants [28,29]. In addition, the promotion of tissue integration is not often tested on antimicrobial screens, and, if it happens to be included, the testing is done separately from the antibacterial properties [30,31,32,33,34]. This is less than ideal as anti-biofilm/antibacterial capability and tissue integration should be preferably tested together. *In vitro* co-culture, models (involving both bacterial and mammalian cells) have been applied to better mimic the clinical situation. These models do not only assess the effects of the leads or materials on bacterial and mammalian cells at the same time but they also provide information on their effect in the interaction between these two cell types [9,35]. 

Our goal in this investigation is to study the applicability of these naturally compound-derived anti-biofilm leads (**DHA1**, **DHA2**, and **FLA1**) on the antimicrobial protection of biomaterials. Biofilm inhibiting effects of these leads were first studied using the collection and clinical bacterial strains. The anti-biofilm effects were then measured on a competitive colonization model of titanium surfaces, based on the protocol developed by Perez-Tanoira et al. [36], exposed simultaneously to *Staphylococcus aureus* strains and human osteosarcoma cells (SaOS-2), as well as with titanium pre-conditioned with media supplemented with serum proteins. Furthermore, as it has been previously shown that the incubation of biomaterials with SaOS-2 cells before implantation can be an effective strategy to prevent bacterial adhesion and biofilm formation [10], we additionally tested if the naturally-derived anti-biofilm leads would enhance this preventive effect given by cellular pre-exposure. 

## 2. Materials and Methods 

### 2.1. Compounds

Two dehydroabietic acid derivatives (**DHA2** and **DHA1**) were synthesized according to [20]. Their spectral data were identical to those reported in [23]. The flavan derivative coded **FLA1**, 6-chloro-4-(6-chloro-7-hydroxy-2,4,4-trimethylchroman-2-yl)benzene-1,3-diol, was purchased from TimTec (product code: ST075672, www.timtec.net). Control antibiotics were purchased from Sigma-Aldrich: rifampicin (13292-46-1) and penicillin G (69-57-8) (coded **RIF** and **PEN**, respectively). 

### 2.2. Bacterial Strains

Bacterial studies were performed with the collection strain *S. aureus* ATCC 25923 (American Type Culture Collection, Manassas, Virginia) and five clinical strains isolated from hip prostheses and osteosynthesis implants at the Hospital Fundación Jiménez Díaz (Madrid, Spain) [37] (*S. aureus* P1, P2, P4, P18, and P61). 

### 2.3. Effects of Compounds on Biofilm Viability in 96-Wells Microplates

Bacteria (*S. aureus* ATCC 25923 and the five clinical strains) were cultured in 30 g/L tryptic soy broth (TSB, Neogen^®^, Lansing, Michigan, USA) under aerobic conditions at 37 °C, 220 rpm for 4 h to reach exponential phase. For forming biofilms, these exponentially grown cultures (10^6^ CFU/mL) were added into flat bottomed 96-well microplates (Nunclon Δ surface, Nunc, Roskilde, Denmark). The anti-biofilm effects of the compounds were assessed prior to biofilm formation and post-biofilm formation. This was carried out as described earlier [17]. Briefly, compounds (at a concentration of 50 µM, 0.25% DMSO) were added simultaneously with the bacterial suspension and effects were examined after incubation at 37 °C, 200 rpm for 18 h, to assess the prevention of biofilm formation. For the post-biofilm formation testing, biofilms were first formed during 18 h (37 °C, 200 rpm), compounds were added, and plates were incubated for 24 h at 37 °C, 200 rpm. Untreated biofilms (only exposed to culture media and 0.25% DMSO), cell-free wells containing only TSB and wells containing biofilms, and 0.25% of DMSO were included as solvent controls. Antibiotics **RIF** and **PEN** were used as positive controls, at the same concentration of the studied compounds. The effects on the biofilm viability were assessed following the protocol of [38] by resazurin staining. Briefly, the biofilms were washed twice (200 µL per well) with phosphate buffered saline (PBS) and stained with 20 µM resazurin for 20 min at room temperature (RT), 200 rpm. The top fluorescence of the reduced resazurin was measured at λ_excitation_ = 560 nm and λ_emission_ = 590 nm using a Thermo Scientific Varioskan LUX Multimode microplate reader.

### 2.4. Selection of The Best Biomass-Producer from the Clinical Strains

In order to assess the differences in biomass production between the clinical strains and select the best strain for the co-culture studies, the biofilm biomass was quantified by crystal violet staining. To do so, after the resazurin measurement, biofilms were fixed with 99.8% MeOH (200 µL per well) for 15 min. After removal of MeOH, wells were allowed to dry and 190 µL of crystal violet (0.0023%, *v*/*v*) was added and cells were stained for 5 min at room temperature (RT), followed by 2 cycles of manual washing (200 µL per well) with milliQ-water. Wells were let dry for 15 min at RT. After this, the crystal violet-bound stain was solubilized in 96% ethanol (200 μL per well). The plates were incubated for 1 h at RT, and the stained biofilm biomass was then quantitated by measuring the absorbance values at 595 nm using a Thermo Scientific Multiskan Sky microplate spectrophotometer. 

### 2.5. Competition Model on A Titanium Surface

#### 2.5.1. Culture of Human Cells

Human osteosarcoma SaOS-2 cells (89050205, European Collection of Authenticated Cell cultures, ECACC) were grown in minimal essential medium (MEM) (Sigma Aldrich, St. Louis, Missouri, USA) supplemented with 10% heat-inactivated fetal bovine serum (FBS) (Sigma Aldrich, St. Louis, Missouri, USA) containing 500 UI/mL penicillin and 0.1 mg/mL streptomycin (Sigma Aldrich, St. Louis, Missouri, USA). Cells were maintained at 37 °C in 5% CO_2_ in a humidified incubator.

#### 2.5.2. Cytotoxicity of Compounds in 96-Well Plates 

Before proceeding with the co-culture studies, the possible cytotoxicity of the compounds to SaOS-2 cells was assessed in opaque-walled well plates, using the Promega CellTiter-Glo^®^ luminescent cell viability assay. This assay is based on the quantification of the ATP present, which signals the presence of metabolically active cells, and therefore allows the calculation of the number of viable cells. To do this, 50,000 cells per well were seeded. They were exposed to a concentration of 50 µM of each compound (0.25% DMSO); cells without treatment were used as control, and effects of DMSO (0.25%) were also measured. Media alone wells (with MEM) were used as blanks, and in the case of **RIF**, a blank containing only this compound was also included, to exclude possible interference related to its red color. After 24 h incubation, Promega CellTiter-Glo^®^ assay was carried out, and the luminescence was measured using a Thermo Scientific Varioskan LUX Multimode microplate reader. 

#### 2.5.3. Culture of Staphylococci and Human Cells

The bacteria *S. aureus* (25923 or the clinical strains) was pre-cultured overnight at 37 °C in 5 mL of TSB medium [39]. It was later centrifuged at 4500× *g* for 10 min, the supernatant was discarded, and the pellet washed three times with PBS. The optical density of the bacterial suspension was measured at λ of 600 nm with a Thermo Scientific Multiskan Sky microplate spectrophotometer according to the McFarland standard. The final concentration of the *S. aureus* suspension was 10^4^ CFU/mL. This suspension was then added to titanium coupons (0.4 cm height, 1.27 cm diameter, BioSurface Technologies Corp., Bozeman, MT, USA), onto which the different tested compounds or the control antibiotics had been added at a concentration of 50 µM and inserted in the different wells of a 24-well plate (Nunclon Δ surface, Nunc, Roskilde, Denmark). 

On the other hand, for the SaOS-2 cells, the media was refreshed 24 h before the experiment started with MEM 10% FBS (without antibiotics), in order to clear out possible traces of antibiotics on the maintenance media. On the day of the experiment the cells were detached with a Trypsin:EDTA solution and re-suspended in MEM 10% FBS; afterwards, they were seeded at a concentration of 10^5^ cells/mL on the titanium coupons, onto which the bacterial suspension had been added as well as the studied compounds and control antibiotics (as described in the previous paragraph). The well plates containing the coupons were maintained in co-culture with a solution containing 10^4^ CFU of *S. aureus* and 10^5^ SaOS-2 cells in a total volume of 1 mL of MEM:PBS 5% FBS for 24 h, as originally described in [36]. Titanium with added **RIF** or **PEN** (both, 50 μM) were used as positive (antibiotic) controls. As co-culture controls, titanium coupons without the addition of the tested compounds or the control antibiotics were exposed to both cellular systems (*S. aureus* and SaOS-2 cells) at the concentrations previously described. In addition, bacterial controls (exposed or not to the compounds, without SaOS-2 cells), SaOS-2 cells controls (exposed or not to the compounds, without bacterial cells), and a DMSO control (0.25 % DMSO coating titanium coupon in 10^5^ SaOS-2 cells/mL), were also included. Furthermore, an additional bacterial control (10^4^ CFU/mL of *S. aureus* on 1 mL of MEM:PBS 5% FBS) was set in order to assess the possible background caused by *S.aureus* in the viability measurement of SaOS-2 cells by luminescence. 

### 2.6. Competition Model on a Titanium Surface Pre-Incubated with SaOS-2 Cells 

Following the protocol of Perez-Tanoira et al. [10], a concentration of 10^5^ SaOS-2 cells/mL was seeded on titanium coupons and incubated for 24 h. After the end of the incubation period, the cell medium was removed and samples were washed three times with sterile PBS to remove any non-adherent human cells. The different tested compounds (**DHA1**, **DHA2**, **FLA1**, **RIF**, and **PEN**) were added to the coupons at a concentration of 50 µM. A suspension containing 10 ^4^ CFU/mL of *S. aureus* and 10^5^ SaOS-2 cells/mL on 1 mL of MEM:PBS 5% FBS was added to each coupon and incubated for 24 h. As a co-culture control, a titanium coupon that had been pre-incubated with the SaOS-2 cells and later added bacterial and SaOS-2 cells (as described before) but not exposed to compounds, was used. As a bacterial control, a suspension of 10^4^ CFU/mL of *S. aureus* on 1 mL of MEM:PBS 5% FBS was added to a titanium coupon without pre-incubation with SaOS-2 cells. As a cellular control, titanium was incubated with 10^5^ SaOS-2 cells/mL on 1 mL of MEM:PBS 5% FBS, without the addition of bacterial cells. Additionally, another bacterial control (10^4^ CFU/mL of *S. aureus* on 1 mL of MEM:PBS 5% FBS ) was set in order to assess the possible background caused by *S.aureus* in the measurement of SaOS-2 cellular viability by luminescence.

### 2.7. Competition Model on a Titanium Surface Pre-Conditioned with Serum-Containing Media 

Following the protocol of Campoccia et al. [40], the titanium coupons were exposed to MEM containing 10% of FBS for 30 min. After the incubation period, the medium was removed and samples were washed once with serum-free MEM. Compound **DHA1** was added to the coupons at a concentration of 50 µM. A suspension containing 10^4^ CFU/mL of *S. aureus* and 10^5^ SaOS-2 cells/mL on 1 mL of MEM:PBS 5% FBS was added to each coupon and incubated for 24 h. As a co-culture control, a titanium coupon that had been pre-conditioned with the 10% FBS MEM and added bacterial cells (as described before) but not exposed to compounds, was used. In addition, a co-culture control with titanium without pre-conditioning with 10% FBS MEM was included. As bacterial controls, a suspension of 10^4^ CFU/mL of *S. aureus* on 1 mL of MEM:PBS 5% FBS was added to a titanium coupon, both non-preconditioned and pre-conditioned with 10% FBS MEM. As cellular controls, titanium was incubated with 10^5^ SaOS-2 cells/mL on 1 mL of MEM:PBS 5% FBS, without the addition of bacterial cells, both non-preconditioned and pre-conditioned with 10% FBS MEM, as in the case of the bacterial controls. Additionally, another bacterial control (10^4^ CFU/mL of *S. aureus* on 1 mL of MEM:PBS 5% FBS) was set in order to assess the possible background caused by *S. aureus* in the measurement of SaOS-2 viability by luminescence.

### 2.8. Measurement of SaOS-2 Cells Viability 

Coupons were washed once with PBS and cells detached with 500 µL of a 1:10 trypsin:EDTA solution. Then, 500 µL of MEM (containing 10% FBS) was added to neutralize the trypsin:EDTA solution. The resulting (1 mL) suspension was centrifuged at 150× *g*. The supernatant was discarded and 100 µL of MEM containing the cellular pellet was transferred to an opaque-walled well plate and the Promega CellTiter-Glo^®^ luminescent cell viability assay was carried out. The possible background signal corresponding to *S. aureus* was subtracted from the co-culture wells 

### 2.9. Bacterial Adherence and Biofilm Formation

Titanium coupons were washed with TBS to remove remaining planktonic cells and then they were transferred into Falcon tubes containing 1 mL of 0.5% (*w/v*) Tween^®^ 20-TSB solution. After that, the tubes were sonicated in an Ultrasonic Cleaner 3800 water sonicator (Branson Ultrasonics, Danbury, CT, USA) at 25 °C, for 5 min at 35 kHz. The tubes were mixed vigorously for 20 s prior to and after the sonication step. Serial dilutions were performed from the resulting bacterial suspensions and plated on Tryptic Soy Agar (TSA, Neogen^®^, Lansing, MI, USA) plates. 

### 2.10. Fluorescence Imaging

Titanium coupons were incubated in the same exact conditions as described in Section 2.5.3 and Section 2.6. After incubation with the different compounds, coupons were manually washed three times with PBS. The dried coupons were stained for 2 min with a rapid fluorescence staining method using acridine orange (BD Diagnostics, Sparks, MD, USA) at a concentration of 0.1 mg/mL. After the staining, coupons were rinsed with sterile water to remove the excess of dye. Images were taken with an Invitrogen EVOS M5000 imaging system (Thermo Scientific, Waltham, Massachusetts, USA)**.** On each coupon, 8–10 fields were viewed and photographed at magnifications of 10x and 20x. Representative images are shown in the results. All imaging experiments were performed in duplicates and experiments were repeated three times. 

### 2.11. Statistical Analysis

The quantitative data are reported as mean of at least 3 technical replicates ± SEM, and all experiments were repeated 3 times. Data were analyzed using GraphPad Prism 8 for Windows. Non-parametric tests were used. For statistical comparisons, Welch’s unpaired *t*-test and one-way ANOVA with Bonferroni correction were applied, and *p* < 0.05 was always considered as statistically significant. 

## 3. Results

### 3.1. Effect on the Prevention and Killing of Biofilms Formed by S. aureus Clinical Strains in 96-well microplates

The three anti-biofilm leads studied here had been earlier tested only using collection strains [18,20]. Thus, prior to starting their effects on biomaterials, we set out to test their anti-biofilm efficacy using five different clinical *S. aureus* strains, isolated from hip prostheses and osteosynthesis implants [37]. These results were compared to the effect of the compounds on *S. aureus* ATCC 25923, the collection strain previously used as a reference. The effects on the biofilm viability were measured using a redox staining assay (resazurin-based) on biofilms grown on the polystyrene surface of 96-well microplates. 

**Figure 2:**
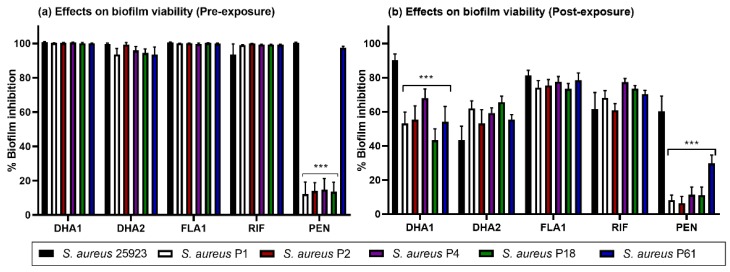
Effect of the two dehydroabietic acid (DHA) derivatives (**DHA1** and **DHA2**), the flavonoid-derivative (**FLA1**), and two control antibiotics (**RIF** and **PEN**), at a concentration of 50 µM, when exposed before biofilm are formed (**a**) or on pre-formed *S. aureus* biofilms (**b**). Biofilms formed by one collection strain (ATCC 25923) and 5 different clinical isolates were tested. Statistical differences are indicated in comparison to the collection strain, *** *p* < 0.001. Results are expressed as mean ± SEM of three technical replicates in experiments repeated three times.

As can be seen in Figure 2a, all three compounds caused over 90% prevention of biofilm formation by all clinical strains, as well as the previously tested reference strain, with an effect fairly similar to **RIF**, one of the used control antibiotics. In contrast, **PEN** (the other control antibiotic tested here) was only efficient against the collection strain and one of the clinical strains (P61). When the effect was measured on pre-formed biofilms of the clinical strains (Figure 2b), the inhibitory effects of the three compounds was reduced (compared to Figure 2a), but, still, a significant inhibitory effect was measured in all cases and it was higher than the one displayed by **PEN**. The second control antibiotic, **RIF,** remained effective and able to cause over 60% inhibition of the viability of the clinical pre-formed biofilms (Figure 2b).

**Figure 3:**
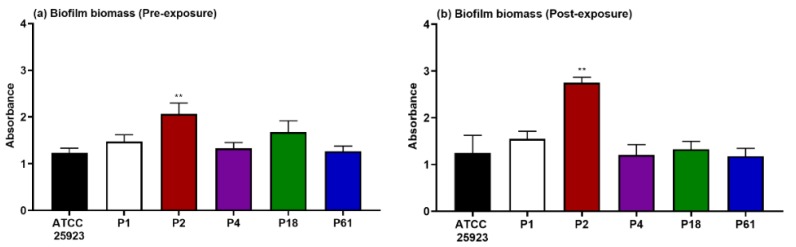
Biofilm biomass produced by *S. aureus* ATCC 25923 in comparison with five clinical strains resulting after 18 h incubation (**a**) and 42 h incubation (**b**). Statistical differences are indicated in comparison to the collection strain, ** p < 0.01. Results are expressed as mean ± SEM of three technical replicates in experiments repeated three times.

For the rest of the studies, one of the *S. aureus* clinical strains, P2, was selected and compared to the effect on the reference collection strain (*S. aureus* ATCC 25923). This decision was based on the fact that this clinical *S. aureus* strain P2 was the one with the highest biomass-containing biofilm when compared to the collection strain *S. aureus* 25923 (Figure 3).

### 3.2. Effect on the prevention of S. aureus Adhesion in a Competitive Colonization Model on Titanium Coupons

Prior to testing the capacity of the compounds on the competitive colonization model, their possible cytotoxic effects towards SaOS-2 cells were assessed on polystyrene 96-well microplates (Figure S1). None of the compounds showed cytotoxicity at a concentration of 50 µM on either of the materials.

**Figure 4:**
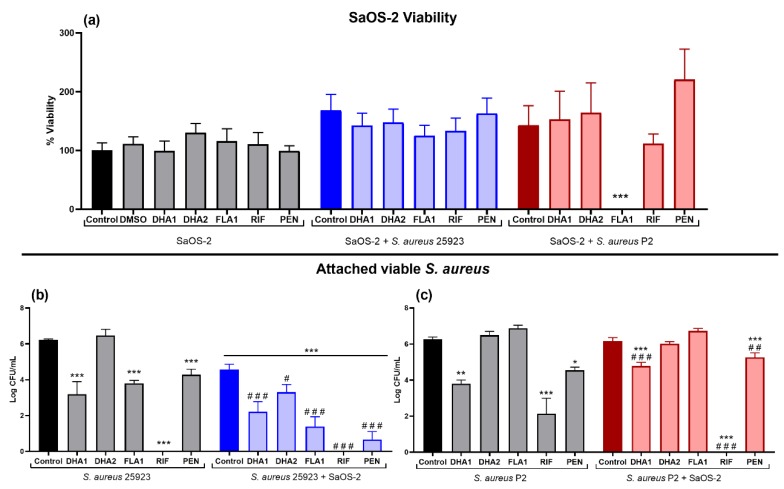
Effect of the two DHA derivatives (**DHA1** and **DHA2**), the flavonoid-derivative (**FLA1**), and two control antibiotics (**RIF** and **PEN**) on the competitive colonization assay performed in titanium coupons. (**a**) Results corresponding to the viability of SaOS-2 cells when cultured on titanium coupons alone (SaOS-2, grey bars), or co-cultured with *S. aureus* ATCC 25923 (SaOS-2 + *S. aureus* 25923, blue bars), or P2 clinical *S. aureus* strain (SaOS-2 + *S. aureus* P2, red bars); (**b**,**c**) Results corresponding to the effects on attached viable *S. aureus* measured when the ATCC 25923 (**b**) or P2 clinical *S. aureus* strain (**c**) was used. Percentage of viability of SaOS-2 cells was calculated with respect to untreated controls after 24-h incubation on titanium coupons, using glow luminescence signal resulting from ATP production by viable SaOS-2 cells. Viable counts (log of CFU/mL) of *S. aureus* ATCC 25923 or the clinical strain P2 were also measured after 24-h incubation on titanium coupons when co-cultured with SaOS-2 cells. “*” represents differences with the corresponding monoculture control and “#” represents differences with the corresponding co-culture controls (* *p* < 0.05; ** *p* < 0.01; *** *p* < 0.001)/(# *p* < 0.05; ^##^ *p* < 0.01; ^###^ *p* < 0.001). Results are expressed as mean ± SEM of three technical replicates in experiments repeated three times.

Next, the effects of the compounds on SaOS-2 viability, as well as on the *S. aureus* 25923 and P2 viable attached cells were measured, when bacteria and human cells were simultaneously co-cultured (Figure 4). The first part of Figure 4a (to the left) shows how none of the compounds caused any inhibition of the SaOS-2 adherence to titanium, in the absence of bacterial cells. The rest of Figure 4a (middle and right panels) show the effects of the compounds on SaOS-2 viability when bacteria and human cells are simultaneously co-cultured. The presence of either the collection (SaOS-2 + *S. aureus* 25923, blue bars) or the clinical (SaOS-2 + *S. aureus* P2, red bars) *S. aureus* strain induced a slight proliferative effect on the SaOS-2 cells, but with no statistical relevance in any of the cases. Only compound **FLA1** significantly diminished SaOS-2 viability in the presence of the clinical strain P2 (Figure 4a; SaOS-2 + *S. aureus* P2). 

On the other hand, the presence of SaOS-2 cells caused a significant reduction of the attached viable *S. aureus* ATCC 25923 when compared with the material incubated only with bacteria (blue control bar versus black control bar in Figure 4b, *p =* 0.007). In addition, all three compounds reduced the number of viable *S. aureus* ATCC 25923 cells in co-cultured with SaOS-2 cells (blue bars), compared to the bacterial control (Figure 4b). Compound **DHA1** managed to cause a 4-log reduction when compared to the bacteria grown in monoculture, and more than a 2-log reduction when compared to the corresponding co-culture control (Figure 4b). The reduction of bacterial adhesion obtained after treatment with compound **DHA2** is slightly smaller but yet significant (*p =* 0.0247), being the number of *S. aureus* ATCC 25923 attached in **DHA2**-treated samples about 1-log less than in the corresponding co-culture control. Compound **FLA1** caused a reduction of the viable *S. aureus* ATCC 25923 comparable to the one obtained with **DHA1** (*p* < 0.001). Both controls antibiotics caused a very significant reduction in the number of viable *S. aureus* ATCC 25923 cells. In fact, **RIF** completely prevented the adherence of this collection strain. 

With respect to the clinical *S. aureus* strain P2, the mere presence of mammalian cells did not cause a reduction of the attached viable bacteria when compared to the bacterial monoculture controls (red control bar versus black control bar in Figure 4c). Compound **DHA1** significantly reduced (*p* < 0.001) the viable attached *S. aureus* P2 by almost 2-log when compared to corresponding bacterial (monoculture and co-culture) controls. Compounds **DHA2** and **FLA1** did not cause any significant reduction of the viable clinical bacteria in this competitive colonization assay. Antibiotics **RIF** and **PEN** significantly reduced the viable attached clinical bacterial strain when compared to both bacterial controls, but **PEN** lost activity, as it was the case with compound **DHA1**.

**Figure 5:**
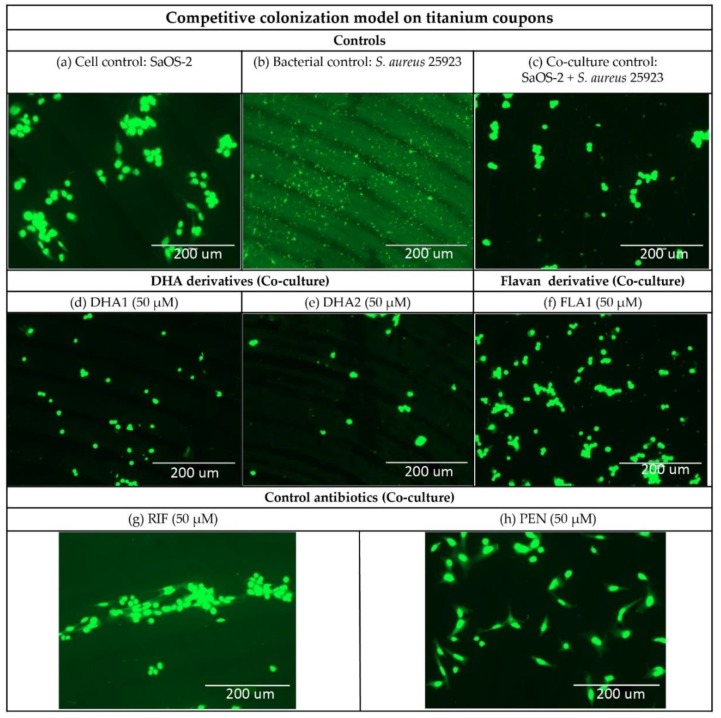
Representative fluorescence microscope images of titanium coupons treated under different conditions in the competitive colonization model. Upper row of the images (Figure 5a–c) correspond to the controls: (**a**) titanium covered by 10^5^ human cells/mL (cell control); (**b**) titanium covered by 10^4^ CFU/mL of *S. aureus* ATCC 25923 (bacterial control), and (**c**) 10^4^ CFU/mL of *S. aureus* ATCC 25923 and 10^5^ human SaOS-2 cells/mL (co-culture control). Middle row of images (Figure 5d–f) correspond to titanium coupons with the two DHA derivatives (**d**) **DHA1** and (**e**) **DHA2**, and the flavonoid-derivative (**f**) **FLA1**, all added at a concentration of 50 µM and co-cultured with *S. aureus* ATCC 25923 and 10^5^ human SaOS-2 cells/mL. The bottom row of images (Figure 5g,h) are representative images of titanium coupons with the two control antibiotics (**g**) **RIF** and (**h**) **PEN**, all added at a concentration of 50 µM and co-cultured with 10^4^ CFU/mL of *S. aureus* ATCC 25923 and 10^5^ human SaOS-2 cells/mL. The samples were stained with acridine orange (BD Diagnostics, Sparks, MD, USA).

Figure 5 shows fluorescence microscope images of the titanium coupons in the competitive colonization model, using the reference collection strain (*S. aureus* ATCC 25923). Figure 5 confirms that the presence of *S. aureus* ATCC 25923 does not cause a reduction in the number of SaOS-2 cells. A slight change in SaOS-2 cell morphology can be noticed when comparing the SaOS-2 monoculture control (Figure 5a) with the co-culture control (Figure 5c). The presence of bacteria seems to cause a slight reduction in the size of the cells and a loosening on its adhesive shape. This change in morphology is not observed in the samples treated with the control antibiotics, given its high antibacterial activity, but it can be noticed in the samples treated with **DHA1**, **DHA2**, and **FLA1** as they do not completely prevent the adherence of bacterial cells. The *S. aureus* ATCC 25923 remaining on those samples might be responsible for these changes observed in SaOS-2 cell morphology.

### 3.3. Effect on the Prevention of S. aureus Adhesion in a Competitive Colonization Model on Titanium Coupons Pre-Incubated with SaOS-2 Cells

**Figure 6:**
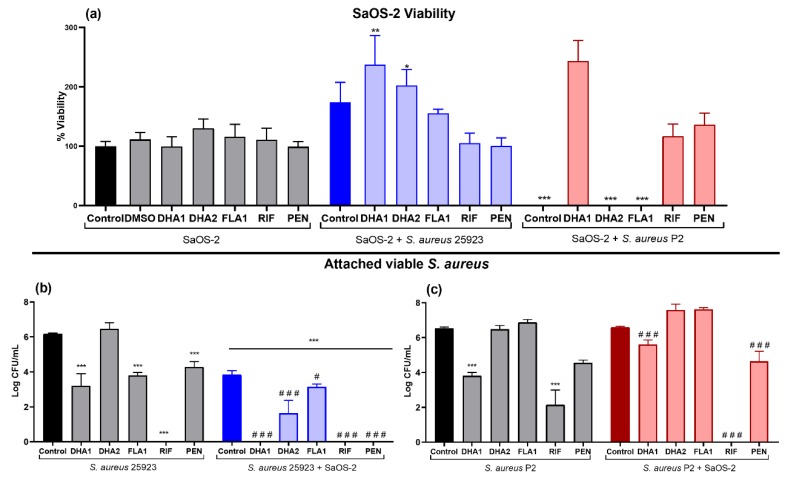
Effect of the two DHA derivatives (**DHA1** and **DHA2**), the flavonoid derivative (**FLA1**) and two control antibiotics (**RIF** and **PEN**) on competitive colonization on titanium coupons pre-incubated for 24 h with SaOS-2 cells. (**a**) Results corresponding to the viability of SaOS-2 cells when cultured on titanium coupons alone (SaOS-2, grey bars), or co-cultured with *S. aureus* ATCC 25923 (SaOS-2 + *S. aureus* 25923, blue bars), or P2 clinical *S. aureus* strain (SaOS-2 + *S. aureus* P2, red bars); (**b**,**c**) Results corresponding to the effects on attached viable *S. aureus* measured when the ATCC 25923 (**b**) or P2 clinical *S. aureus* strain (**c**) was used. Percentage of viability of SaOS-2 cells was calculated with respect to untreated controls after 24-h incubation on titanium coupons, using glow luminescence signal resulting from ATP production by viable SaOS-2 cells. Viable counts (log of CFU/mL) of *S. aureus* ATCC 25923 or the clinical strain P2 were also measured after 24-h incubation on titanium coupons when co-cultured with SaOS-2 cells. “*” represents differences with the corresponding monoculture control and “#” represents differences with the corresponding co-culture controls (* *p* < 0.05; ** *p* < 0.01; *** *p* < 0.001)/(^#^ *p* < 0.05; ^##^ *p* < 0.01; ^###^ *p* < 0.001). Results are expressed as mean ± SEM of three technical replicates in experiments repeated three times.

Figure 6 shows the effects of the compounds in coupons pre-incubated with SaOS-2 cells when using both the reference *S. aureus* collection (ATCC 25923, blue bars) or the clinical strain (P2, red bars). 

With respect to the effect on the SaOS-2 cells, only when *S. aureus* ATCC 25923 and SaOS-2 cells were simultaneously incubated with compounds **DHA1** or **DHA2**, a proliferative effect was seen (*p =* 0.004 and 0.037, respectively) compared to the corresponding control (Figure 6a, middle panel, SaOS-2 + *S. aureus* 25923). In contrast, co-culture of the clinical isolate P2 caused a highly significant decrease (*p* < 0.001) on mammalian cell viability (Figure 6a, SaOS-2 + *S. aureus* P2). In the controls, mammalian cells did not survive when co-cultured in the presence of *S. aureus* P2 strain, neither did the SaOS-2 cells treated with the compounds **DHA2** and **FLA1**. However, a significant proliferative effect of SaOS-2 cells was found in the presence of compound **DHA1**. Exposure to both control antibiotics also prevented the bacterial-induced cytotoxicity and resulted in the protection of SaOS-2 cells, similarly to compound **DHA1** (Figure 6b, red bars, SaOS-2 + *S. aureus* P2). 

Figure 6b,c shows the effects of the compounds on the viable attached bacteria when using coupons pre-incubated with SaOS-2 cells. The pre-incubation of titanium with SaOS-2 cells significantly reduced the number of attached ATCC 25923 *S. aureus* strain (blue control bar versus gray control bar in Figure 6b, *p* < 0.001). Exposure to compound **DHA1** as well as the control antibiotics caused a total reduction of *S. aureus* ATCC 25923 attachment (Figure 6b, *p* < 0.001 in all cases). Compounds **DHA2** and **FLA1** caused a reduction of the viable attached bacteria, when compared to the bacterial (ATCC 25923) control (*p* < 0.001, in both cases), and the reduction was also significant when compared to the cellular pre-coated coupons (control of *S. aureus* 25923 + SaOS-2, Figure 6b) (*p* < 0.001 and *p =* 0.035, respectively).

In contrast, the results of Figure 6c show how the positive impact of cellular pre-conditioning was not detected for the clinical *S. aureus* strain P2. Interestingly, exposure to compound **DHA1** significantly reduced 1-log the viable attached P2 strain when compared to both the bacteria control and the control corresponding to the pre-coating of titanium with SaOS-2 cells (SaOS-2 + *S. aureus* P2 strain) (*p* < 0.001). The activity of **RIF** was preserved against the clinical strain, while **PEN**, despite losing some activity when compared to its effect against the collection strain, also significantly reduced the viable bacteria (P2 strain) attached (*p* < 0.001 in both cases). 

**Figure 7:**
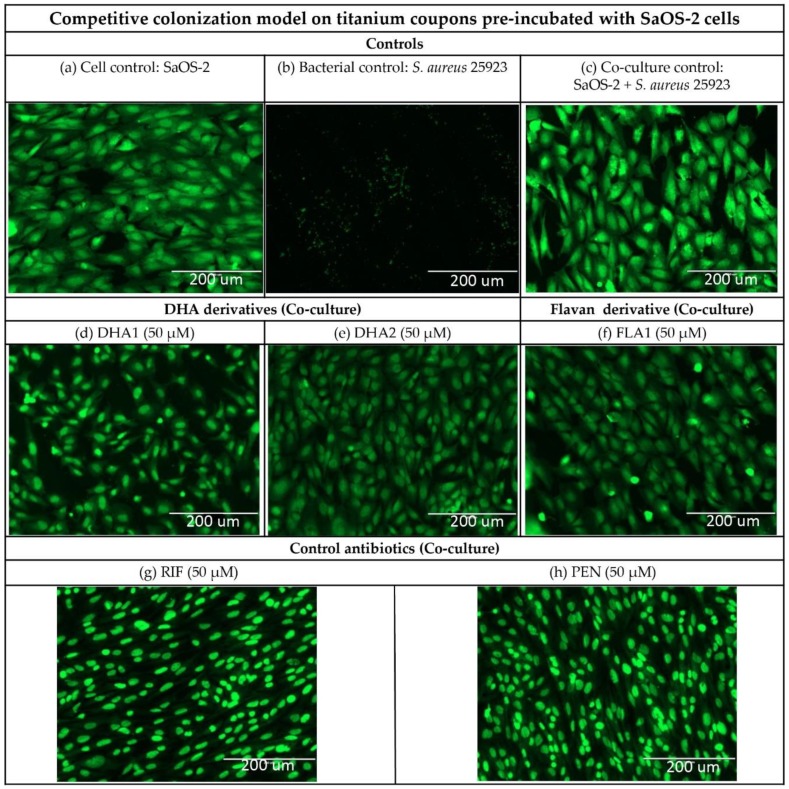
Representative fluorescence microscope images of titanium coupons treated under different conditions in a competitive colonization model with cellular pre-coating. Upper row of the images (Figure 7a–c) correspond to the controls: (**a**) titanium covered by 10^5^ human cells/mL (cell control); (**b**) titanium covered by 10^4^ CFU/mL of *S. aureus* ATCC 25923 (bacterial control), and (**c**) 10^4^ CFU/mL of *S. aureus* ATCC 25923 and 10^5^ human SaOS-2 cells/mL (co-culture control). Middle row of images (Figure 7d–f) correspond to titanium coupons with the two DHA derivatives (**d**) **DHA1** and (**e**) **DHA2**, and the flavonoid derivative (**f**) **FLA1,** all added at a concentration of 50 µM and co-cultured with *S. aureus* ATCC 25923 and 10^5^ human SaOS-2 cells/mL. The bottom row of images (Figure 7g,h) are representative images of titanium coupons coated with the two control antibiotics (**g**) **RIF** and (**h**) **PEN**, all added at a concentration of 50 µM and co-cultured with 10^4^ CFU/mL of *S. aureus* ATCC 25923 and 10^5^ human SaOS-2 cells/mL. The samples were stained with acridine orange (BD Diagnostics, Sparks, MD, USA).

Figure 7 shows fluorescence microscope images of the titanium coupons in the competitive colonization model using titanium coupons that had been pre-coated with SaOS-2 cells. The protective effect of the pre-coating with SaOS-2 cells is visible, as the cells do not show the morphology change that was observed when the SaOS-2 cells were directly co-cultured with *S. aureus* ATCC 25923 (Figure 5c versus Figure 7c). When compared to the cellular monoculture control (Figure 7a), the different treatments (Figure 7d–h) do not appear to affect the morphology or the number of SaOS-2 cells. 

### 3.4. Effect on the Prevention of S. aureus Adhesion in a Competitive Colonization Model on Titanium Coupons Pre-Conditioned with Media Containing High Serum Concentration

**Figure 8:**
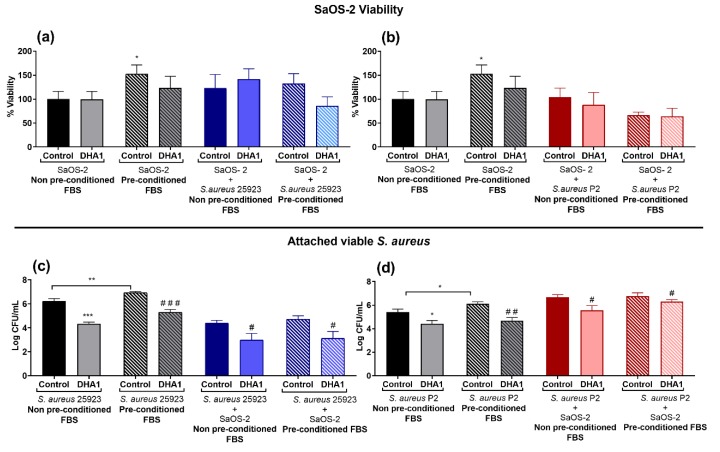
Effect of the DHA derivative **DHA1** on the competitive colonization assay performed in titanium coupons pre-conditioned with media supplemented with FBS. (**a**,**b**) Results corresponding to the viability of SaOS-2 cells when ATCC 25923 (**a**, blue bars) or P2 clinical *S. aureus* strain (**b**, red bars) were used. (**c**,**d**) Results corresponding to the effects of attached viable *S. aureus* measured when ATCC 25923 (**c**, blue bars) or P2 clinical strain (**d**, red bars) were used. The percentage of viability of SaOS-2 cells was calculated with respect to untreated controls after 24-h incubation on titanium coupons, using glow luminescence signal resulting from ATP production by viable SaOS-2 cells. Viable counts (log of CFU/mL) of *S. aureus* 25923 and the clinical strain P2, respectively, were also measured after 24-h incubation on titanium coupons when co-cultured with SaOS-2 cells. “*” represents differences with the control in mono-culture exposed to the non-preconditioned titanium coupon (SaOS-2 non- preconditioned with FBS/ *S. aureus* 25923 non-preconditioned/*S. aureus* P2 non-preconditioned). “#” represents differences with the control of the subgroups (* *p* < 0.05; ** *p* < 0.01; *** *p* < 0.001)/(^#^ *p* < 0.05; ^##^ *p* < 0.01; ^###^ *p* < 0.001). Results are expressed as mean ± SEM of three technical replicates; experiments repeated three times.

Figure 8 shows the effects of **DHA1** on SaOS-2 viability, as well as on the attachment of *S. aureus* ATCC 25923 and P2 viable cells when bacteria and human cells are simultaneously co-cultured in titanium that has been pre-conditioned with media containing 10% FBS. In parts (a) and (b) of Figure 8, it can be seen how SaOS-2 adheres better to the titanium surface when it is pre-conditioned with media containing 10% FBS (*p* = 0.0387). However, this enhanced adherence is not observed when SaOS-2 cells are co-cultured with bacteria, nor with the collection strain (Figure 8a) or the clinical strain (Figure 8b). In turn, in Figure 8c,d, it can also be observed how both bacterial of strains adhere significantly more to the titanium surface pre-conditioned with media containing 10% FBS. (Figure 8c, *p* = 0.0063; Figure 8d, *p* = 0.0313). 

In the case of the collection strain (Figure 8c), it can be again observed how the presence of SaOS-2 cells decreased the attachment of S. aureus (*p* < 0.001). The pre-conditioning with FBS does not make a difference in bacterial adherence when bacterial cells are co-cultured with SaOS-2 cells (Figure 8c). Exposure to the compound **DHA1** significantly reduced the attached *S. aureus* ATCC 25923 when compared to the corresponding controls of the subgroups: *S. aureus* 25923 non-preconditioned FBS; *S. aureus* 25923 preconditioned FBS; *S. aureus* 25923 + SaOS-2 non-preconditioned FBS, and *S. aureus* 25923 + SaOS-2 preconditioned FBS (*p* < 0.001; *p* < 0.001; *p* = 0.026, and *p* = 0.019, respectively). As for the clinical P2 strain (Figure 8d), the effect observed in Figure 4c is repeated, the presence of SaOS-2 cells does not decrease the adherence of the *S. aureus* P2 strain. The pre-conditioning with media containing 10% FBS does not influence the adherence of *S. aureus* P2 when co-cultured with SaOS-2 cells (Figure 8d). Once more, the compound **DHA1** significantly reduced the attached *S. aureus* P2 strain when compared to the corresponding controls of the different subgroups: *S. aureus* P2 non-preconditioned FBS; *S. aureus* P2 preconditioned FBS; *S. aureus* P2 + SaOS-2 non-preconditioned FBS, and *S. aureus* P2 + SaOS-2 preconditioned FBS (*p* = 0.017; *p* = 0.0011; *p* = 0.027, and *p* = 0.025, respectively).

## 4. Discussion

In this study, the applicability of three previously discovered antimicrobial compounds to be incorporated into implanted medical devices that would prevent the formation of *S. aureus* biofilms was studied. To examine such effects, a competitive model was utilized, allowing us to investigate, in a more realistic scenario, the implantation of a biomaterial, which provides a substratum to host either tissue–cell integration or bacterial colonization. These two phenomena are in conflict because, after the adherence of either one, the surface is less prone to colonization by the other. Tissue integration and bacterial contamination of medical devices have been extensively studied as independent phenomena [41]. However, only recently, experimental assays have been established in which fair competition can be investigated during the development of new biomaterial-coating strategies [36,42]. Using such tools, it has been recently shown that the incubation of typical implant materials with human SaOS-2 cells before implantation represents an innovative and effective way to reduce the bacterial living space available and prevent *S. aureus* adhesion, thus protecting biomaterials against biofilm formation [10]. Such an approach would offer an attractive concept that could be further enhanced by the presence of antimicrobial compounds.

In the current study, we hypothesized that two DHA derivatives (**DHA1** and **DHA2**) and a flavan derivative (**FLA1**), which our group had previously reported as promising anti-biofilm leads [18,20], could find applicability in the protection of medical devices against infections. Here, we started by evaluating their antimicrobial efficacy against clinical bacterial strains [37]. In the case of *S. aureus*, its origin is particularly relevant. Besides being a causative agent of implant infections, *S. aureus* is part of the normal bacterial flora of human skin and mucosal surfaces. One of the most relevant virulence factors expressed by *S. aureus* is the cell wall-anchored (CWA) protein, surface proteins covalently attached to peptidoglycan that significantly influence the colonization ability and posterior survival of the bacteria in the host, which is particularly relevant in medical devices infections [43]. Among these, the microbial surface component recognizing adhesive matrix molecules (MSCRAMM) facilitates the adherence of the bacteria to the host tissue; in the case of bone implants, the capacity of *S. aureus* to interact with collagenous proteins might be important in the pathogenesis of possible osteomyelitis [44]. This makes it obvious that the inter-strain virulence, and as a consequence, the efficacy of the tested compounds, may drastically change [28]. Our results show how that, despite being very efficient at both preventing and killing biofilms formed by the reference *S. aureus* ATCC 25923 strain, the antibiotic **PEN** loses a great part of its activity when tested against most of the clinical strains (all except P61, Figure 2a,b). On the contrary, compounds **DHA1**, **DHA2**, **FLA1**, as well as the control **RIF**, kept their preventive activity against all the clinical strains (Figure 2a). All of the tested compounds were also able to inhibit at least 30% of the viability of already formed biofilms by both the reference collection and the clinical strains. It was also observed differences in the biomass quantity produced by different clinical strains, which could represent an additional virulence factor that varies depending on the strain. In Figure 3, it can be seen how *S. aureus* P2 produces more biomass than the collection strain. This can influence the treatment and clearance of these clinical biofilms, in comparison with the collection strain. 

Moreover, efficacy in preventing biofilm formation of the different compounds was tested on titanium, given its relevance as biomaterial used for orthopedic implants. In Figure 4, it can be seen that compound **DHA1** preserved its antimicrobial activity when tested on titanium. However, compound **DHA2** was shown to lose its prevention capability on this material. This change is even more evident with the clinical strain, where neither the compound **DHA2** nor **FLA1** managed to prevent biofilm formation (Figure 4c). 

Titanium treated with **DHA1**, **DHA2**, or **FLA1** was studied in a competitive colonization model with both bacteria and human cells. We used an *S. aureus* concentration of 10^4^ CFU/mL as more than 10^2^ CFU of *S. aureus* is necessary to establish a prosthesis infection and 10^4^ CFU/mL in surgery without an implant. On the other hand, it has been proposed that using concentrations higher than 10^6^ CFU/mL might be questionable and not relevant from a clinical perspective [45,46]. According to present results, and in concordance with the ones reported by [36], the presence of *S. aureus* did not significantly affect the viability of SaOS-2 cells (Figure 4a). Previously, Yue et al. [47] have shown that low bacterial concentrations increase cellular adhesion, likely as a result of the stress response caused by bacteria on the mammalian cells, which are then forced to compete more effectively and withstand cellular detachment. This effect was observed in both the collection strain and the clinical strain. None of the compounds produced a negative effect on SaOS-2 cellular viability, but surprisingly, the cells treated with the compound **FLA1** died when exposed to the clinical *S. aureus* P2 strain (Figure 4a). From fluorescence imaging (Figure 5), it can be seen how the presence of bacteria had an effect on the morphology of SaOS-2, despite not affecting the number of attached cells. It can be observed that this change in morphology is reduced in presence of **RIF** (Figure 5g), likely due to the fact that this antibiotic reduces practically to zero the presence of *S. aureus* ATCC 25923 viable cells.

As mentioned before, the success of an anti-infective prosthesis relies not only on the efficacy to eradicate bacteria but also in its ability to promote bone-implant osseointegration. Titanium is an inert material that does not accelerate this process, which is why many approaches towards implant development aim at bio-functionalizing titanium to improve its bioactivity. The difficulty remains in modifying the titanium in such a way that it promotes osseointegration while being antimicrobial, as on many occasions, the antimicrobial agents incorporated are cytotoxic to the osteoblasts. Nie et al. [48] developed a biofunctionalized titanium with bacitracin that proved to be antimicrobial and cytocompatible in vitro. The efficacy of such titanium on preventing infection and improving osteoinductivity was later proven *in vivo* [49]. This is a good example of how an antimicrobial that is intended to form part of a prosthesis has not only to be proven *in vitro* as antimicrobial but also cytocompatible, as both qualities are essential for correct integration of the prosthesis. The *in vitro* system described in this study not only assess both qualities in the same assay, but it also gives information on the effect that the antimicrobial has on the adherence of the material of each cell type, mammalian and bacterial, when they are present together.

The second part of this study aimed at assessing the utility of the compounds in enhancing the positive effect of pre-incubating materials with human SaOS-2 cells [10]. As previously demonstrated, the development of an infection would highly depend on what type of cells colonize the surface of an implant first, the cells of the host or the invading bacteria cells. Unfortunately, during surgery, bacterial cells frequently are in advantage as they can be introduced onto an implant before integration with host tissue even starts, and they have a faster replication rate. Giving advantage to the host cells would facilitate tissue integration and diminish the risk of bacterial infection [9]. By combining the protective effect of pre-conditioning titanium with mammalian cells with the previously reported anti-biofilm capability of the studied compounds, it was expected to accomplish a drastic reduction of the biofilm formation.

The pre-exposure of SaOS-2 to *S. aureus* 25923 generates the same slight proliferation effect on the mammalian cells at 24 h (Figure 6 in comparison to Figure 4). This proliferation is less acute in the case of **RIF** and **PEN**, probably due to their high efficacy of killing the bacterial cells. None of the compounds appeared to affect the morphology of the cells. Perez-Tanoira et al. [10] reported a drop in the proliferation of SaOS-2 cells at 48 h exposed to the same concentration of the clinical isolate *S. aureus* 15981. In our study, the presence of the clinical *S. aureus* strain P2 dramatically decreases SaOS-2 cell viability in co-culture, which may be explained by a higher virulence of this strain. This drop on the cell viability can also be observed in the groups treated with **DHA2** and **FLA1**, probably due to the lack of antibacterial activity (Figure 6a).

It has to be taken into account, that when implanting a medical device, the first event occurring, before the race for the surface starts, is the interaction of the implant’s surface with physiological fluids and the subsequent adsorption of host proteins. The adhesion of contaminating bacteria usually occurs once the implant is protein-coated. The adhesive glycoprotein fibrinogen is a major plasma glycoprotein. At sites of trauma and injury, fibrinogen serves as a major host substrate for *S. aureus* adhesion, as this bacterium expresses fibrinogen-biding proteins [44]. For this reason, we tested the pre-conditioning of titanium with fibrinogen at a physiological concentration (1g/L) [50], following the protocol of Opperman et al. [51]. There were no differences in the adherence of *S. aureus* to a titanium surface pre-conditioned with fibrinogen when compared to the non-conditioned surface (Figure S2). These results are in line with the ones obtained by Rao et al. [52] that showed how the pre-conditioning of titanium with fibrinogen and fibrinogen clotted with thrombin promoted osseointegration, but the biofilm formation by *Enterococcus faecalis* in these bio-functionalized materials did not show significant differences with the titanium control.

Next, we also tested the influence of pre-conditioning the titanium surface with media supplemented with a higher concentration of FBS, in the same competition model, as it contains other major proteins present in the blood, such as albumin, which can also be adsorbed by titanium [53,54]. We demonstrated that the adherence of bacteria is higher when the surface is pre-conditioned in this manner, and this bacterial enhancement occurs in both bacterial strains (Figure 8c,d). Nevertheless, mammalian cell adhesion to the preconditioned surface seemed also to increase, which is the reason why the results obtained in this part of the study are very similar to the ones obtained in the competition model without pre-conditioning. The compound **DHA1** prevented bacterial adherence of both the collection and clinical *S. aureus* strains also on pre-conditioned titanium (Figure 8c,d). 

In this study, we analyzed three compounds that, based on our earlier results, were regarded as promising candidates to prevent *S. aureus* biofilms related infections. However, out of the three, we demonstrated here that only compound **DHA1** remains effective in conditions that are more likely to be encountered during an *in vivo* bacterial infection in an orthopedic implant. In the case of the compound **DHA2**, its lack of activity might be given by the fact that the concentration used here is slightly below its MIC [20]. As with most antimicrobials, a common concern is unspecific cytotoxicity. Both **DHA** derivatives had already been tested on mammalian cells, specifically HL cells (originating from the human respiratory tract). Compound **DHA1** did not cause any reduction in the viability of this cell line, but with a concentration of 100 µM of **DHA2**, only 23% of the cells remained viable. This is why a concentration of 50 µM was chosen, as in a 96-well microplate system, it did not show cytotoxic effect but kept a considerably strong anti-biofilm capability. An increase of the concentration on the co-culture system would have probably shown a higher antimicrobial effect, but also a cytotoxic one, so it would have anyway limited the efficacy of **DHA2** as a possible implant coating. On the contrary, the compound **FLA1** was used in a dosage that was expected to be effective [18], and despite the effectiveness shown in 96-well microplates, the lack of activity in the competitive colonization system indicates that it is most likely not suitable for protection of implants.

In this study, the compound **DHA1** was shown to be a promising candidate to form part of a bone implant, based on the results of the competitive colonization model. However, this co-culture system used here is not meant to replace *in vivo* experimentation, and it can still be further improved, for instance, by utilizing primary osteoblast cells. It has been shown how the results obtained in a co-culture system utilizing osteoblast cells differ from the ones obtained with SaOS-2 cells [36]. The co-culture using SaOS-2 is very convenient (methodologically-wise), but it is to be kept in mind that because SaOS-2 is a sarcoma cell line, it has a faster division rate, and can be in general more resilient to adverse conditions. Utilizing autologous osteoblasts of the patient would be the ideal scenario to avoid possible rejection of the prosthesis. The co-culture model could also be further enriched by the addition of immune cells, such as neutrophils, which are also present at the moment of implantation. In any case, as it stands, the co-culture model of *S. aureus* and SaOS-2 cells does offer a deeper insight into the protective capacity of investigational antimicrobial compounds, particularly those intended for the protection of bone implants.

These results highlight the importance of developing new protocols *in vitro* that would more effectively select the best antimicrobial candidates for biomedical applications, and that would minimize the translational gap between *in vitro* screening data and the *in vivo* clinical scenario.

## 5. Conclusions

We concluded that one DHA derivative, **DHA1**, would be a promising candidate for the coating of biomaterials in order to prevent biofilm formation. This compound shows a preventive activity of *S. aureus* biofilm formation by both collection and clinical strains, and it prevents *S. aureus* adhesion to titanium surfaces, while also favoring the adhesion of mammalian cells to titanium.

## Supplementary Materials

The following are available online at https://www.mdpi.com/2076-2607/8/3/345/s1, Figure S1: Effect of the two DHA derivatives (**DHA1** and **DHA2**), the flavonoid derivative (**FLA1**) and two control antibiotics (**RIF** and **PEN**) on SaOS-2 viability when cultured in 96 wells polystyrene plates. Figure S2. Viable counts of *S. aureus* (ATCC 25923 and clinical strain P2) after 24 h incubation on titanium coupons and titanium coupons pre-conditioned with 1 g/L of fibrinogen.
