# Supplementary material for: Strategies to Prevent Biofilm Infections on Biomaterials: Effect of Novel Naturally-Derived Biofilm Inhibitors on a Competitive Colonization Model of Titanium by Staphylococcus aureus and SaOS-2 Cells
Source: Microorganisms. 2020 Feb 29;8(3):345. doi: 10.3390/microorganisms8030345 (PMC7143544; doi:10.3390/microorganisms8030345)
Supplement: Supplementary file 1 [file microorganisms-08-00345-s001.pdf]

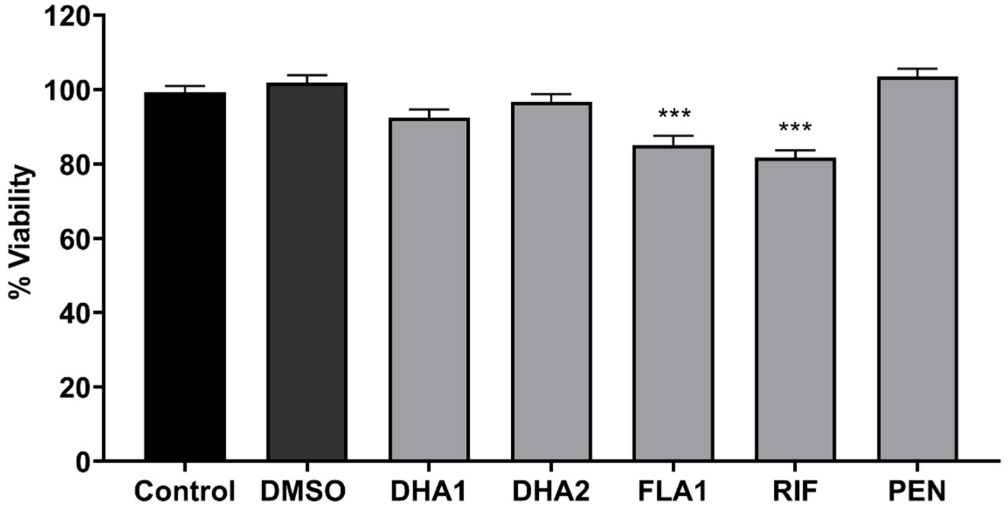

Figure S1. Effect of the two DHA derivatives (DHA1 and DHA2), the flavonoid-derivative (FLA) and two control antibiotics (RIF and PEN) on SaOS-2 viability when cultured in 96 wells polystyrene plates. Compounds were tested at a concentration of 50 μM. The viability percentage was calculated with respect to untreated controls after 24 h incubation. "*" indicates differences with the control (p*<0.05; p**<0.01; p***<0.001). Results are expressed as mean ± SEM of three technical replicates, experiments repeated three times.

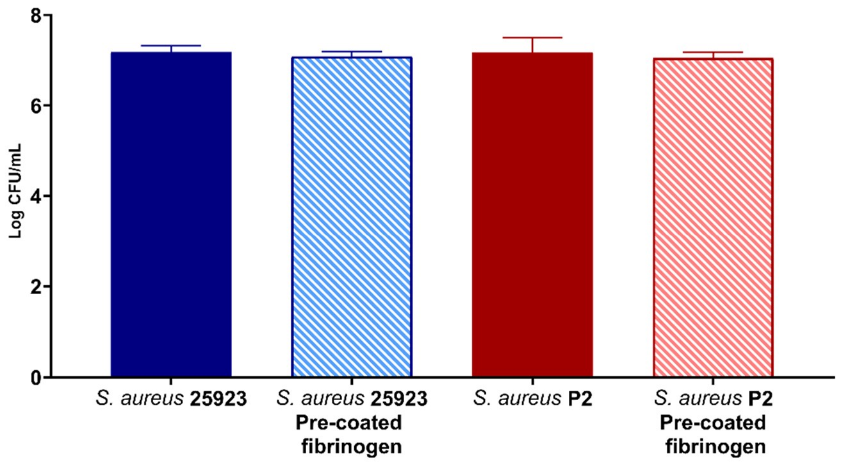

Figure S2. Viable counts of *S. aureus* (ATCC 25923 and clinical strain P2) after 24 hours incubation on titanium coupons and titanium coupons pre-conditioned with 1 g/L of fibrinogen. Results are expressed as mean ± SEM of two technical replicates, experiments repeated two times.
